# Quantitative Lung Perfusion Scintigraphy in Patients with Congenital Heart Disease

**DOI:** 10.4103/1995-705X.76802

**Published:** 2010

**Authors:** Ahmed Fathala

**Affiliations:** King Fasial Specialist Hospital and Research Center, Riyadh, Saudi Arabia

**Keywords:** Pulmonary blood flow, congenital heart disease, lung perfusion, scintigraphy

## Abstract

The objectives of this article are to review different patterns and potential pitfalls of quantitative lung perfusion scintigraphy (LPS) in patients with congenital heart disease (CHD). The patterns of quantitative LPS in patients with CHD include normal symmetrical bilateral perfusion to both lungs, unilateral absent perfusion in one lung, unilateral decreased perfusion, and multiple segmental perfusion abnormalities that suggest pulmonary embolism. Knowledge of several potential pitfalls is very important to avoid false interpretations; common pitfalls are related to type of site of injection (upper versus lower extremities), right or left upper extremity in case of persistence of left superior vena cava and previous surgery. An important incidental finding that may prompt immediate attenuation is multiple segmental defect that suggests asymptomatic pulmonary embolism, which is relatively common in this population.

## INTRODUCTION

In complex congenital heart disease (CHD), it is important to know the differential lung perfusion because asymmetrical lung perfusion is one of the predictors of the outcome and exercise capacity.[1] Quantitative lung perfusion scintigraphy (LPS) is considered the gold standard for quantitative evaluation of pulmonary perfusion in most patients with CHD.[2,3] The indications for quantitative LPS in patients with CHD may be classified as pre- and post-surgical and/or percutaneous intervention.

Quantitative LPS studies are simple to perform. No specific patient preparation is necessary.[4] Technetium-99m (^99^Tc) macroaggregated albumin (MAA) is the radiopharmaceutical used. The recommended dose of ^99m^TC is 37–148 MBq (1–4 mCi). With the aid of computer software, region of interests (ROIs) are placed over the right and left lung and the geometric mean of the anterior and posterior counts in each region is calculated. Each lung can be further divided into thirds to create superior, middle and inferior ROIs. This division does not correlate with anatomic division into lobes.

### Normal perfusion lung scan

Patients with CHD with normal pulmonary artery have normal perfusion with essentially equal perfusion to both lungs [Figure 1a and b]. In the anterior view, cardiac silhouette and aortic knob are commonly identified. Commonly, a cardiac silhouette larger than expected from chest radiograph may be produced by hypermobility of the heart when lateral images are obtained. Perfusion lung scan should be correlated with chest X-ray for accurate evaluation of cardiac size. A rare but potential pitfall in the interpretation of perfusion lung scan in CHD patients with symmetrical bilateral pulmonary artery stenosis may have symmetrical relative perfusion in both lungs.

**Figure 1a:**
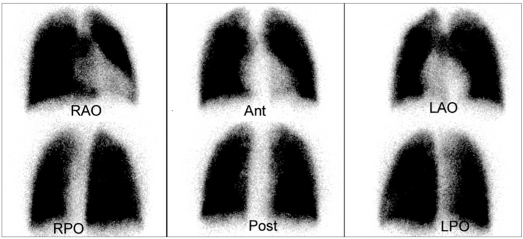
Normal quantitative lung perfusion scintigraphy (LPS) in a 16-year-old male with transposition of great arteries and S/P arterial switch operation. Standard six projections LPS show normal and symmetrical perfusion in both lungs

**Figure 1b:**
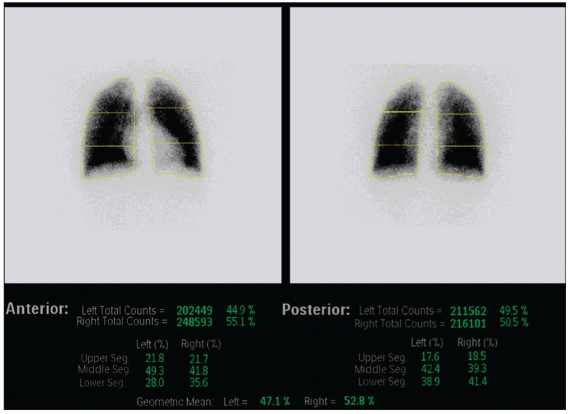
Normal quantitative lung perfusion scintigraphy (LPS) in a 16-year-old male with transposition of great arteries and S/P arterial switch operation. Quantitative perfusion analysis reveals that the right lung contributes to 53% and the left lung contributes to 47% of the total pulmonary perfusion

### Unilateral absence of lung perfusion

Unilateral absence of lung perfusion is seen in patients with congenital absence of pulmonary artery, wherein the ipsilateral lung perfusion occurs through collaterals from bronchial arteries that cannot be assessed by lung perfusion except in patients with functioning right to left shunt. In addition, unilateral absence of pulmonary artery perfusion is seen in many CHD patients, such as patients with Tetralogy of Fallot TOF and absent right or [Figure 2a and b] left pulmonary artery.[5] Other differential diagnoses of unilateral absence of lung perfusion include pulmonary aplasia (absence of ipsilateral pulmonary artery, absence of ipsilateral pulmonary tissue and bronchus terminates in dilated blind pouch), pulmonary embolism [Figure 3a and b] and hyperlucent lung syndrome.

**Figure 2a:**
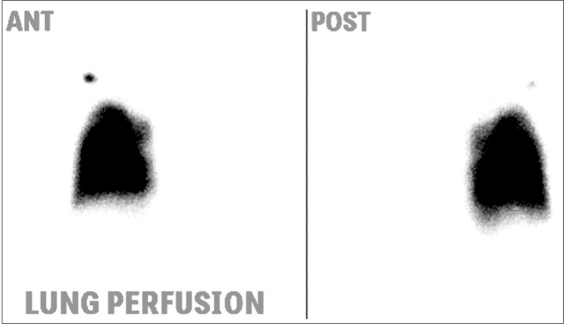
Total absence of the left lung perfusion in an 11-year-old male with fontan. Quantitative lung perfusion scintigraphy shows no perfusion in the left lung

**Figure 2b:**
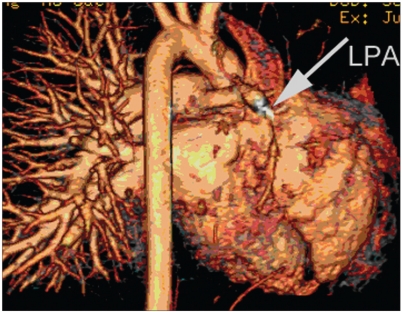
Total absence of the left lung perfusion in an 11-year-old male with fontan. The 3D volume magnetic resonance angiography (MRA) reveals hypoplastic and totally occluded left pulmonary artery (LPA) (arrow)

**Figure 3a:**
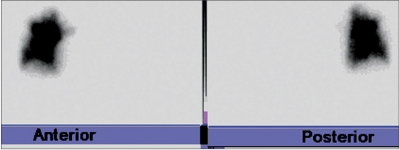
Total absence of the left lung perfusion in a 35-year-old female with severe pulmonary hypertension. The perfusion is totally absent in the left lung. In addition, there are multiple peripheral subsegmental perfusion abnormalities consistent with pulmonary embolism

**Figure 3b:**
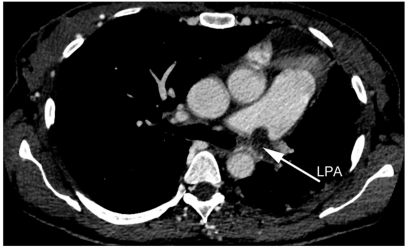
Total absence of the left lung perfusion in a 35-year-old female with severe pulmonary hypertension. Pulmonary computed tomography angiography (CTA) reveals complete occlusion of LPA with thrombus (arrow)

### Unilateral decreased lung perfusion

Unilateral decreased relative lung perfusion is the most common perfusion abnormality seen in patients with CHD. The etiology of unilateral decreased lung perfusion can be divided into an isolated congenital branch PA stenosis and branch pulmonary artery (PA) stenosis seen in many CHD patients, such as TOF with right or left PA stenosis, transposition of the great arteries (s/p arterial switch operation) and truncus arteriosus.[6] Quantitative LPS studies may be used before the surgery or intervention to assess the severity of the stenosis. Post-intervention LPS is used for follow-up [Figure 4a and b].

**Figure 4a:**
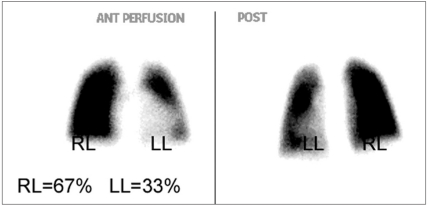
A 7-year-old female with TOF and LPA stenosis. Before LPA stent, the left lung contributes to 33% and the right lung contributes to 67% of the total lung function

**Figure 4b:**
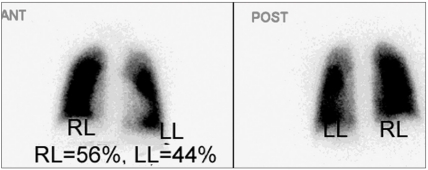
A 7-year-old female with TOF and LPA stenosis. Interval improvement of left lung perfusion after LPA stent, the left lung perfusion increases to 44%

### Right to left shunt

Where there is a right to left shunt, some of the MAA escape from the pulmonary circulation and lodge in the systemic capillaries.[7] By estimating the counts in the lungs and in the systemic circulation, it is possible, after an intravenous injection of MAA, to estimate the size of the right to left shunt [Figure 5 a and b]. Presence of aortopulmonary collaterals blood vessels (APCs) may cause significant systemic to pulmonary shunt.[8] APCs are associated with a variety of CHD (TOF and pulmonary atresia). Right to left shunt may occur in many types of CHD with elevated right ventricular pressure, such as TOF, transposition of great arteries, truncus arteriosus and ebstein anomaly. Differential diagnosis of abnormal right to left shunt includes hepatic cirrhosis and pulmonary arteriovenous malformation.

**Figure 5a:**
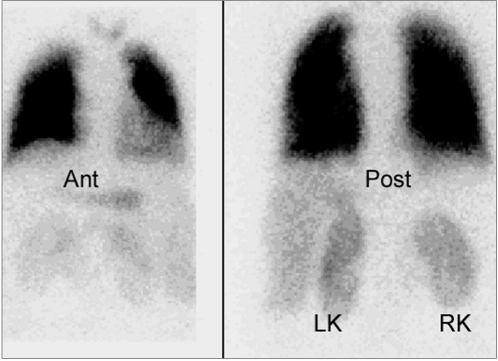
Right to left shunt in a 10-year-old female with atrial septal defect (ASD) and ventricular septal defect VSD. The perfusion is normal and symmetrical in both lungs. Abnormal activity is seen in both kidneys (left kidney and right kidney). This finding indicates right to left shunt

**Figure 5b:**
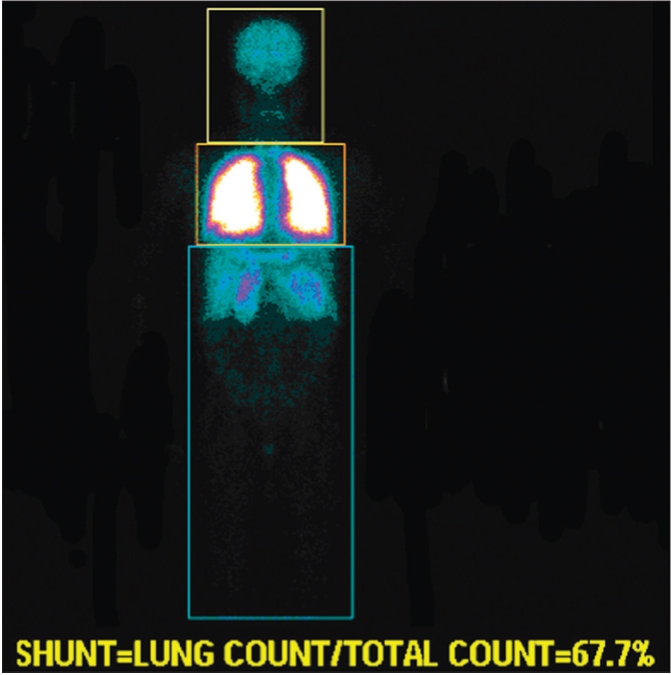
Right to left shunt in a 10-year-old female with ASD and VSD. To quantify right to left shunt, region of interests were drawn around the entire body and around the lung. Right to left shunt is determined by subtracting the count in the pulmonary region from the whole body region. In this case, the right to left shunt was 32%

### Multiple segmental perfusion abnormalities (Pulmonary embolism)

**Figure 6:**
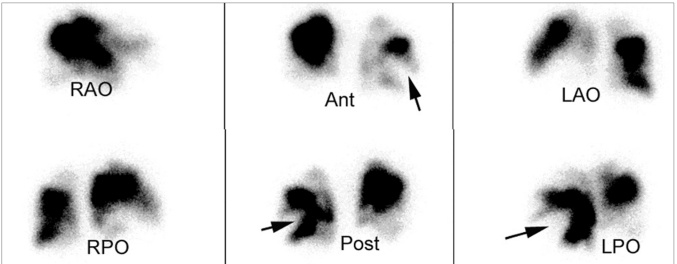
This patient is a 17-year-old female with history of congenital heart disease. The lung perfusion scintigraphy shows multiple, large, segmental, wedge-shaped, perfusion defects in both lung fields (arrows), with greater involvement of the right lung than the left lung

Thrombosis and thromoboembolism can be a significant cause of morbidity and mortality after Fontan operation [Figure 6]. The possible risk factors that may contribute to thrombosis in patients with CHD include low flow state, stasis in venous pathways, right to left shunt, blind cul de sacs, prosthetic materials and/or arrhythmias.[9] It has been demonstrated that the prevalence of silent pulmonary embolism in adult Fontan patients is 17%; initial screening was performed with ventilation perfusion lung san and, subsequently, was confirmed with computed tomography (CT) pulmonary angiogram. Although LPS was performed without a ventilation scan, findings of multiple segmental perfusion defects should raise the possibility of pulmonary embolism and further investigation such as CT pulmonary angiogram must be considered.

### Radiation exposure, pitfalls and limitations

LPS lung scan imaging is associated with a radiation dose of about 1 mSv, equivalent to 50 frontal CXR. Repeat investigation of this exam can therefore contribute significantly to radiation burden for children and adolescents. In patients with atriopulmonary anastomoses, total or partial cavopulmonary connection (Fontan circulation), LPS does not optimally reflect the genuine perfusion ratio because the geometry of pulmonary arterial anastomoses with superior vena caval and inferior vena caval pathways leads to abnormal streaming and distribution of blood flow to branch pulmonary arteries.[10] Injection into the arm will lead to preferential deposition of MAA into the left lung via a surgical superior vena cava – right pulmonary artery SVC-RPA shunt, but injection into the leg leads to a more even distribution. Preferential left lung perfusion has been reported in patients with persistent left SVC and stenosis at the site of anastomoses at Blalock shunt. In addition, LPS tends to underestimate left lung perfusion in patients with inequality of pulmonary regurgitation.[11]

### Cardiac magnetic resonance imaging as an alternative technique to measure differential pulmonary perfusion

Magnetic resonance (MR) imaging is now increasing in the assessment of CHD in both children and adults.[12] Quantitative blood flow has been measured using phase-contrast magnetic resonance (PC-MR). PC-MR has shown to be accurate in adult patients where pulmonary artery is often normal. Recent studies have demonstrated that PC-MR is able to assess both anatomic and quantitative information in patients with CHD with complex pulmonary artery anatomy and regurgitation.[10] The main advantage of MR is that it avoids ionizing radiation and comprehensive examination of anatomy, morphology and flow quantification in one exam.

The limitations to MR are several, which include: (1) absolute contraindications as in the case of pacemaker, (2) the need for general anesthesia and (3) several potential errors, such as incorrect prescription for location or angle, incorrect technical parameters and signal dephasing owing to turbulent flow in case of discrete stenosis.

## SUMMARY

It is important to measure the differential lung perfusion in patients with CHD as it predicts the outcome and exercise capacity. Quantitative LPS is simple, safe and widely available. Several patterns of LPS are seen in patients with CHD. Knowledge of different patterns, potential pitfalls and limitations are very important to avid false interpretation with subsequent patient mismanagement. PC-MR imaging may be considered as an alternative test in some patients.
